# The gut takes it all: Enteroendocrine control of developmental growth

**DOI:** 10.1371/journal.pbio.3003921

**Published:** 2026-08-12

**Authors:** Julia B. Cordero

**Affiliations:** 1 School of Cancer Sciences, Wolfson Wohl Cancer Research, University of Glasgow, Glasgow, United Kingdom; 2 CRUK Scotland Institute, Glasgow, United Kingdom

## Abstract

How do developing animals sense nutritional shortages and adjust their growth accordingly? A new study in PLOS Biology shows that the developing intestine is a central regulator of a multi-organ signalling coordinating animal growth in response to microbiome composition and nutritional deficiency.

## Teaser

How developing animals sense nutritional shortages to adjust growth? A new study in *PLOS Biology* shows that the developing intestine is a central regulator of animal growth in response to microbiome composition and nutritional deficiency.

The ability of organisms to tailor their growth to environmental resources is fundamental to survival, reproductive fitness, and evolutionary success. In a new study published in *PLOS Biology*, Bai and colleagues uncover a previously unrecognized role of the developing intestine as a central regulator of a multi-organ signaling, coordinating animal growth in response to microbiome composition and nutritional stress [1]. The authors show that the gut-derived peptide hormone Limostatin (Lst), produced by a small subset of enteroendocrine cells restricted to the anterior midgut of *Drosophila melanogaster* larvae, acts as a nutrient-sensitive hormone that suppresses brain-derived insulin-like peptide 2 (dILP-2) during malnutrition, thereby slowing development and enhancing survival under adverse nutritional conditions.

**Fig 1:**
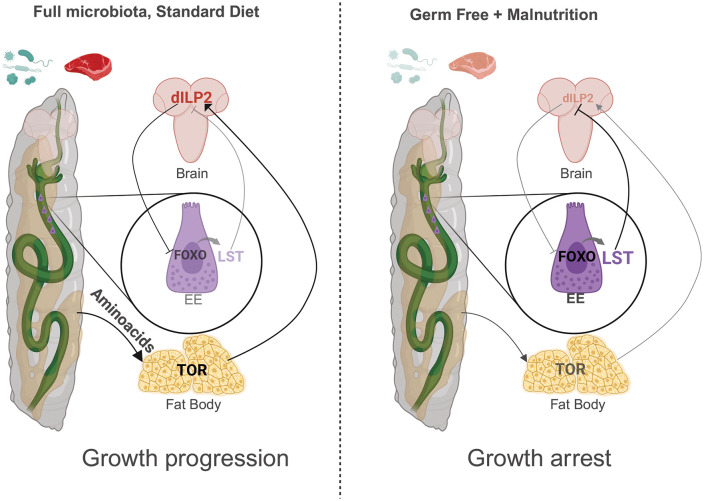
Enteroendocrine-derived Limostatin regulates nutrient-dependent systemic growth in germ-free developing *Drosophila melanogaster.* Protein scarcity sensed by the fat body, impairs dILP-2 production from the brain, and activates FoxO expression in enteroendocrine cells. Foxo induces Lst expression and secretion from enteroendocrine cells to further suppresses circulating dILP-2 and stall growth. Lst, Limostatin; EE, enteroendocrine cells; dILP-2, *Drosophila* insulin-like peptide 2. Created in BioRender. Cordero, J. (2026) https://BioRender.com/6ps04mj.

In their study, Bai and colleagues provide compelling genetic evidence that gut-derived Lst acts as a brake on growth under nutrient-limited conditions [1]. Mechanistically, nutrient scarcity sensed by the fat body—the insect functional equivalent of the liver and adipose tissue—reduces dILP-2 production by the brain, leading to activation of the transcription factor Foxo in enteroendocrine cells. Foxo subsequently induces cell autonomous Lst expression and secretion from enteroendocrine cells, which further suppresses circulating dILP-2 (Fig 1), reinforcing the organism’s growth-restraining response to nutritional challenge. Loss of Lst function from enteroendocrine cells accelerates developmental progression during malnutrition, whereas overexpression delays development. These effects are restricted to protein-deprived animals, indicating that Lst is not a constitutive suppressor of growth but rather a nutrient-dependent regulator of adaptive organismal growth.

Limostatin’s role as a nutrient-dependent insulin signaling inhibitor has been previously reported in adult *Drosophila* [2]. However, in adults, Lst is produced by the corpora cardiaca (CC), a gut-extrinsic endocrine organ that also secretes the glucagon-like hormone, Adipokinetic hormone (AKH). Moreover, Lst secretion from the CC is regulated specifically by dietary carbohydrates. The current study therefore reveals a strikingly distinct developmental role for this hormone, in which Lst production appears to be tuned primarily to protein availability. This difference may reflect an evolutionary adaptation to the heightened demand for amino acids during larval growth.

Enteroendocrine cells in the adult insect and mammalian intestine are well-known mediators of systemic homeostasis upon direct nutrient sensing [3]. Surprisingly, however, rather than directly sensing amino acids through classical intracellular nutrient-sensing pathways, Lst-expressing enteroendocrine cells appear to respond to systemic nutritional status through an inter-organ signaling relay originating in the fat body (Fig 1). Whether this represents a special feature of a subtype of enteroendocrine cells or a global characteristic of the developing enteroendocrine system, remains to be addressed.

Discoveries made through this study significantly expand our understanding of the exquisite richness and specialized nature of the intestinal enteroendocrine system. While the gut has long been recognized as a site of nutrient absorption, growing evidence suggests it also plays an active role in systemic physiological regulation through hormone secretion [3–9]. This study demonstrates that, as in adults [7], a small population of enteroendocrine cells in the larval midgut can sense nutritional stress and coordinate whole-body responses through long-range hormonal communication.

In summary, this fascinating study uncovers a previously unrecognized gut–brain endocrine axis that enables developing animals to adapt to nutritional stress. Importantly, it establishes the developing intestine as an active player in the regulation of systemic growth. More broadly, the work highlights, once more, how genetically tractable model organisms can be leveraged to uncover sophisticated mechanisms that safeguard development under challenging environmental conditions.

Several important questions emerge from this study and represent promising avenues for future research. First, what receptor mediates the developmental actions of Lst? Temporal specialization appears to be critical, as the receptor previously proposed to mediate Lst function in adults [2] does not seem to mediate actions of the developmental hormone. Second, what underlies the selective sensitivity of Lst-producing enteroendocrine cells to systemic insulin signals? Addressing this question will likely require deeper cellular and molecular characterization of the full repertoire of enteroendocrine cells within the larval midgut. Such studies may reveal previously unappreciated diversity and levels of specialization of the enteroendocrine system of the developing intestine. Finally, given the growing appreciation of gut-derived endocrine signals in vertebrate physiology [3,8], conceptual advances from this work may have implications beyond insects. While no direct human ortholog of Lst has been identified, the decretin neuropeptide Neuromedin U (NMU) has been proposed as a potential functional counterpart [2]. If comparable mechanisms exist in mammals, they could reveal new principles governing juvenile growth, nutritional adaptation, and developmental resilience in response to environmental challenges.

## Abbreviations

AKH: Adipokinetic hormone
CC: corpora cardiac
dILP-2: *Drosophila* insulin-like peptide 2
Lst: Limostatin
